# Ethical requirements for musculoskeletal research involving human subjects

**DOI:** 10.1007/s10195-015-0371-x

**Published:** 2015-08-21

**Authors:** Luca Pierannunzii

**Affiliations:** Gaetano Pini Orthopaedic Institute, P.zza C. Ferrari, 1, Milan, Italy

## Introduction

Musculoskeletal research is mostly clinical and participation of human subjects is extremely common. It shares most features with all the surgical research fields, with studies on operative procedures largely prevailing over those on pharmacological interventions. In the orthopedic research scenario, prostheses and other implantable hardware often correspond to the role that drugs play in medical research, with no less potential harm for the populations studied. Not rarely, vulnerable categories are involved, for instance children in pediatric orthopedics. In the emerging branch of musculoskeletal regenerative medicine, embryonic stem cells are not used, but only adult somatic stem cells, which considerably reduces but does not remove the ethical issues connected with these new applications.

Given these premises, guaranteeing human subjects protection is mandatory for authors, editors and publishers, firstly because “the health of my patient will be my first consideration” (from the World Medical Association Declaration of Geneva [1]), but also because biomedical publishing is not exempt from possible litigation. Since editors are ultimately responsible for what is published, they have to enforce strict requirements to ensure that proper ethical standards are fulfilled. Sometimes such requirements seem to subject authors to a burden of paperwork that equals the scientific effort, but intolerance of this is generated by a misinterpretation: science and ethics cannot stand alone, nor can they be neatly separated in any research project.

There are three milestones of ethical human research: the Declaration of Helsinki, informed consent and ethics committee approval. Most journals, as we do at *Journal of Orthopaedic Traumatology*, ask authors to make a precise statement under their own responsibility to guarantee that these requirements are met. But formally reporting adequate ethical standards cannot be considered sufficient for publication, if anywhere along the process of manuscript review relevant concerns are raised about the substantive ethical conduct of the investigators.

## Defining the boundaries of “research involving human subjects”

In most cases connected with musculoskeletal research and publishing, the definition of study involving human participants is obvious and unquestionable. However, we ought to consider some special cases in which this assignment might be debatable.

In certain legislative scenarios, the first part of the definition, “research”, might be questioned. As we will discuss later, in UK service evaluation, audit and public health surveillance are not considered research as they are not meant to produce new generalizable knowledge [2]. These studies may then be conducted with less formal requirements than properly defined research studies.

However, the most interesting grey area is represented by the second part of the definition, “involving human subjects”. Does research based on human specimens, cells, cell lines or simple data involve human subjects? The regulation differs between countries. In the USA we find the clearest answer: human subjects are involved if specimens, cells or data are obtained from living individuals by the investigators through direct intervention/interaction or, alternatively, if investigators obtain identifiable private information linked to the items. This means that cadaver studies and all studies in which repositories like biobanks and databases provide the investigators with coded items without releasing the key that permits them to ascertain the identity of the living subjects to whom data/cells/specimens pertain, do not involve human subjects [3]. The EU regulation does not address the issue with equal clarity. The Recommendation Rec(2006)4 of the Committee of Ministers to member states on research on biological materials of human origin [4] states that research on identifiable biological materials should be performed only with the specific consent of the person concerned, while research on unlinked anonymized biological materials is permitted if no donor’s restriction is violated. But the same recommendation requires that anonymization be assessed by an appropriate review procedure, which means, in other words, that to be prudent all studies on biological materials are equated to human research. However, since the recommendation is not legally binding, researchers should refer to their own national legislation for specific requirements.

## The Declaration of Helsinki

The Declaration of Helsinki was adopted by the 18th World Medical Association General Assembly in 1964 and recently amended in 2013 [5]. It states the “Ethical Principles for Medical Research Involving Human Subjects”, including protecting the life, health and privacy of all patients or healthy subjects, guaranteeing voluntary and informed participation, and requesting an external ethical review of the research projects and the appropriate registration of clinical trials in a public database in order to minimize the risk of selective reporting of results and to avoid unnecessary duplication of research projects. According to the International Committee of Medical Journal Editors (ICMJE), registration is mandatory for all prospective studies, comparative or not, that expose the subjects to a health-related intervention (i.e. surgical procedure or drug administration) and evaluate its outcome. The International Clinical Trials Registry Platform (ICTRP) [6] lists the registries that have been tested and approved by the World Health Organization and accepted by the ICMJE. ClinicalTrials.gov is equally accepted as a data provider to ICTRP.

When authors state, under their own responsibility, that “the study conforms to the Declaration of Helsinki”, they actually guarantee that all the above principles were observed. The following two statements about informed consent and ethics committee approval might be considered pleonastic, as they are already entailed by adherence to the Declaration. However, these additional claims mean to substantiate the ethical conduct of the study through objective documentation, available upon request by the journal, while the assertion to have followed the Helsinki principles might rely subjectively on the authors’ judgement.

## Informed consent

The Council for International Organizations of Medical Sciences (CIOMS) International Ethical Guidelines for Biomedical Research Involving Human Subjects [7] clearly state that all the human subjects enrolled in a study must provide a voluntary informed consent (Guideline 4); it should be obtained after a thorough explanation of the objects and methods, as well as of the possible risks and benefits, giving the subject the chance to revoke their adherence at any time throughout the study. In pediatric orthopedics, the consent must be expressed by the parents (or by any other legally authorized representatives), but if the child’s age allows adequate comprehension, their opinion should be sought and not swayed by the parents’ judgement, namely in the case of refusal (CIOMS Guideline 14). The informed consent should be expressed in writing, and the signed consent form should be stored by the investigators. Exceptionally, other types of documentation can be accepted (i.e. recording or witnessing). The documents should be made available to the editors upon request.

Waiver of the informed consent is a rare occurrence, which must always be provided by the competent ethics committee (EC). When a study involves no more than extracting data from medical records and no potential harm to the studied population is determined, and anonymity and confidentiality are properly safeguarded, such as in retrospective chart reviews, the ethics committee may decide to waive the informed consent, if the study might not be feasible otherwise. Any waiver of or alteration to the formal consent requirements provided by the responsible EC must be reported in the Ethical Standards statement.

A different consent form should be obtained from patients when identifying information is published (i.e. clinical pictures in which anonymization cannot be reliably performed without distorting the scientific meaning of the figure); this is a consent to publish identifiable information about the subject, who should be made aware of what kind of material will be displayed and should accept the corresponding privacy violation.

## Ethics committee (EC) or Institutional Review Board (IRB)

CIOMS ethical guideline 2 requires that “all proposals to conduct research involving human subjects must be submitted for review of their scientific merit and ethical acceptability to one or more scientific review and ethical review committees” [7]. This statement implies that investigators have to obtain clearance from an external and independent body of reviewers before any kind of research project is started. Ethics committees are designed to include members with different fields of expertise, in order to carry out both the scientific and the ethical review; a research protocol with poor scientific basis is a waste of resources and might involve unjustified harm to the studied population, and thus is as ethically unacceptable as a study whose protocol shows an unbalanced risk–benefit ratio.

The organization of ECs varies across countries: in Europe there are national, regional, local and institutional Research Ethics Committees, whose responsibility is determined by local laws. The new Clinical Trials Regulation EU No 536/2014 [8], expected to apply no earlier than May 2016, should favor the harmonization of clinical trials authorization in the European Union, but is not supposed to interfere with the national organization of ECs. Researchers should then continue to refer to their national legislation. In Italy, for instance, ECs were recently reorganized by Regions to comply with the standard of one Committee per million inhabitants, and are made up of about 20 members in position for 3 years. Additional Ethics Committees were also identified in scientific hospitalization and care institutions (IRCCS) [9]. In the USA the corresponding entity is named an Institutional Review Board (IRB), is represented by at least five members with different backgrounds to cover the area of research, is set up at the level of medical and academic institutions, and is regulated by the Office for Human Research Protections (OHRP) within the Department of Health and Human Services (HHS). Commercial or independent IRBs from for-profit organizations are also in existence and equally acceptable, as they are subjected to the same US Federal Rules [3].

Obtaining clearance from the responsible EC/IRB implies that an external body of reviewers, independent from the investigators, has verified that the research is scientifically sound, that the potential risks to the subjects involved are minimized and reasonable in relation to the anticipated benefits, that the participants are equitably selected and thoroughly informed, that consent is properly obtained, and that privacy and confidentiality are protected as well as the welfare and rights of special vulnerable categories.

Exemption from EC/IRB approval is considered in certain cases under special legislation. In the UK, for example, the Health Research Authority waives the ethical review for projects on service evaluation (designed to measure the standards of care provided by a health service), clinical audit (designed to test a service against a predetermined standard) and public health surveillance (designed to manage outbreaks). All these projects are considered outside the scope of research, as they do not create new generalizable knowledge [2]. Since the intent of such projects is rarely the publication of scientific papers, it seldom occurs but, in that event, especially if the paper is submitted to international or non-UK journals, the editors might question the missing EC/IRB clearance. We at *Journal of Orthopaedic Traumatology* adopt the recommendation of the Committee on Publication Ethics (COPE) [10] and consider the paper acceptable if no ethical concerns can be raised. Similarly US Federal Rule 45 CFR 46 states that “Research involving the collection or study of existing data, documents, records, pathological specimens, or diagnostic specimens, if these sources are publicly available or if the information is recorded by the investigator in such a manner that subjects cannot be identified, directly or through identifiers linked to the subjects” is exempt from the Basic HHS Policy for Protection of Human Research Subjects, and may then avoid IRB approval [3]. To avoid misunderstandings that might compromise the acceptance of the manuscript, in such cases authors are advised to: (1) seek the judgement of an IRB/EC representative about exemption instead of making the decision by themselves; (2) cite the national legislation permitting the exemption in the Ethical Standards statement; (3) provide a careful description in Materials and Methods of the procedures meant to minimize the risks to health, privacy and confidentiality of the subjects involved.

## Ethical review and Editors’ responsibility

As the protocol evaluation by the EC/IRB cannot neatly separate scientific and ethical assessment, similarly the manuscript evaluation occurring in any peer-reviewed journal ought to consider both these aspects before expressing a final decision on acceptance or rejection. In most journals, such as in *Journal of Orthopaedic Traumatology*, the first manuscript screening by the Editorial Office already checks the conformity to the ethical requirements reported in the Instructions for Authors. This is merely a formal evaluation, aimed at avoiding misunderstandings and oversights in the preparation of the Ethical Standards statement. The second line of ethical review is represented by peer review itself. Not rarely the referees raise concerns or ask for clarification about ethical issues connected with the study protocol. Such an attitude should be encouraged and promoted. The third line of ethical review is commonly performed by editors, who are ultimately responsible for whatever is published in their journals. Formal conformity to the main ethical requirements (adherence to the Declaration of Helsinki, informed consent, EC/IRB approval) does not guarantee a positive assessment. In doubtful cases the editor may ask the authors to provide protocol documentation, informed consents, EC/IRB reports, or simply more methodological details to form their own judgement about the conduct of the research. In exceptionally critical cases they are entitled to contact the EC/IRB, or even to ask the authors to obtain an additional external review.

Safeguarding the health, welfare, privacy and confidentiality of research participants is a heavy responsibility for editors, and rejection on ethical grounds is a rare but sometimes necessary occurrence, as unpopular among authors who receive it as it is among editors who have to determine it. In some cases the reasons for this extreme decision lie in poor protocol planning, in a limited knowledge of rules and legislation, or simply in underestimating the ethical issues against the scientific ones. However, most ethical concerns arising throughout the review process are merely formal and could be reasonably solved in advance with a careful preparation of the Ethical Standard statement and a detailed description of the risk minimization procedures within the Materials and Methods section. Should further questions be raised, authors are encouraged to provide the journal with all the available information and documentation for an appropriate ethical assessment. To every wise editor, the value of a substantively ethical study will always prevail over a minor noncompliance with formal requirements, as long as the authors cooperate with the journal to elaborate and clarify.

## Final recommendations for authors

The “golden triad” of ethical human research—conformity to the Declaration of Helsinki, informed consent, EC/IRB clearance—is the fundamental Ethical Standards statement that authors should be ready to make for most musculoskeletal research manuscripts. Whenever an exemption from the second or the third requirement is obtained, the local rules or laws allowing the exemption or the EC decision about consent waiver should be reported. Lastly, all the critical issues regarding the protection of the human participants should be clearly addressed in Materials and Methods, as they are at least as relevant as the scientific issues in evaluating the global quality of the research and its eventual publishability.
